# Equity in action: The Diagnostic Working Group of The Undiagnosed Diseases Network International

**DOI:** 10.1038/s41525-024-00422-y

**Published:** 2024-07-05

**Authors:** Elizabeth Emma Palmer, Helene Cederroth, Mikk Cederroth, Angelica Maria Delgado-Vega, Natalie Roberts, Fulya Taylan, Ann Nordgren, Lorenzo D. Botto

**Affiliations:** 1 Discipline of Paediatrics and Child Health, School of Clinical Medicine, Faculty of Medicine and Health, University of New South Wales, Sydney, NSW Australia; 2 Centre for Clinical Genetics, Sydney Childrens’ Hospitals Network, Sydney, NSW Australia; 3 Wilhelm Foundation, Stockholm, Sweden; 4 Department of Molecular Medicine and Surgery, Center for Molecular Medicine, Karolinska Institutet, Stockholm, Sweden; 5 Department of Clinical Genetics and Genomics, Karolinska University Hospital, Stockholm, Sweden; 6 Institute of Biomedicine, Department of Laboratory Medicine, University of Gothenburg, Gothenburg, Sweden; 7 Department of Clinical Genetics and Genomics, Sahlgrenska University Hospital, Gothenburg, Sweden; 8 Division of Medical Genetics, Department of Pediatrics, University of Utah, Salt Lake City, Utah USA

**Keywords:** Molecular medicine, Genetic testing

## Abstract

Rare diseases are recognized as a global public health priority. A timely and accurate diagnosis is a critical enabler for precise and personalized health care. However, barriers to rare disease diagnoses are especially steep for those from historically underserved communities, including low- and middle-income countries. The Undiagnosed Diseases Network International (UDNI) was launched in 2015 to help fill the knowledge gaps that impede diagnosis for rare diseases, and to foster the translation of research into medical practice, aided by active patient involvement. To better pursue these goals, in 2021 the UDNI established the Diagnostic Working Group of the UDNI (UDNI DWG) as a community of practice that would (a) accelerate diagnoses for more families; (b) support and share knowledge and skills by developing Undiagnosed Diseases Programs, particularly those in lower resource areas; and (c) promote discovery and expand global medical knowledge. This Perspectives article documents the initial establishment and iterative co-design of the UDNI DWG.

## Introduction

In 2021, the United Nations (UN) Resolution “Addressing the challenges of persons living with a rare disease and their families” was ratified by all 193 UN Member States^1^. This act, resulting from the concerted global advocacy of patients, their families and supporters, clinicians, and researchers, brought into the global spotlight the challenges faced by the 300 million people living with rare and undiagnosed diseases^2,3^.

The Resolution calls on all Member States to accelerate efforts towards achieving universal health coverage by 2030, including access to diagnostics^1^. The first step to improving the rare disease journey is a timely and accurate diagnosis^4^. Without a diagnosis, improving health and social outcomes is challenging. A diagnosis ends what many families call their ‘diagnostic odyssey’ and provides a firmer basis for more personalized and precise care, including targeted treatment, surveillance, and preventive measures, access to research opportunities including clinical trials, and avoiding unnecessary tests and procedures. A firm diagnosis empowers patients and families and allows them to establish connections with specific rare disease advocacy groups, which, in combination with appropriate health literacy resources, helps them gain access to disability, educational, and employment supports that are suitable for their needs^5,6^. Without a diagnosis, people living with rare diseases often face uncertainty, isolation, and even the stigma that they do not have a ‘real’ condition^7^.

### Barriers to an accurate and timely rare disease diagnosis

To date, over 7000 rare diseases have been recognized, of which an estimated >70% are genetic^8,9^. However, many people with rare diseases, over 50% according to some authors^10^, remain undiagnosed. Regardless of whether this statistic is accurate, multiple barriers to a timely and accurate diagnosis are evident.

The first barrier is recognition. Many clinicians struggle to recognize that their patient’s combination of symptoms and signs may be an individually rare disease, leading to delays or misdiagnoses^11,12^. Only after clinicians have ‘joined the dots’ can these patients undergo appropriate diagnostic investigations or referral to a specialist diagnostician^12^. Strategies to tackle this barrier include global awareness campaigns such as Rare Disease Day, and new clinical education initiatives to increase knowledge about rare diseases, precision diagnostics, precision medicine, and personalized care, and grow the skills of multidisciplinary teamwork, tolerance of uncertainty, and patient-centered and indeed partnered care including genuine listening and curiosity^11–14^.

The second barrier is access to skilled diagnostic teams and appropriate testing. Skilled diagnosticians and appropriate diagnostic tests are scarce resources (when even present), especially in low- and middle-income countries^15^. In addition, the cost of modern sequencing technologies, though decreasing in general, is still relatively high. Moreover, appropriate and informative testing requires a complex infrastructure that includes bioinformaticians, molecular biologists, and clinical experts. In many countries, patients can only access diagnostic tests by paying out of their own pockets^16,17^. There is also an urgent need to facilitate data sharing and to find safe and legal ways to store and share genomic data^18^.

The third barrier, perhaps less obvious but not less impactful, may be socio-cultural. Religious and cultural belief systems can influence the conceptualization and access to a rare disease diagnosis^19^. For example, illness may be interpreted as a trial from God or a recompense for sins^20,21^. This conceptualization can lead people with rare diseases to avoid or delay seeking medical consultation, to prefer alternative care, and to avoid connecting to support groups. Partnerships with community leaders and institutions are critically important to build trust and understanding of the potential benefits of integrating rare disease diagnostics and care in a culturally safe and appropriate manner. An example of the success of such an approach is the upskilling of community-based nurses, doctors, and pharmacists, who already have trusted relationships with community leaders, in genomic medicine through the African Genomic Medicine Training Initiative (AGMT)^22^.

The final barrier is a knowledge gap. Current knowledge of the underlying causes of rare conditions is increasing but still limited^23^. Current clinical diagnostic genomic testing protocols, including analysis and reporting guidelines, are designed to robustly detect and report the ‘low hanging fruit’ of pathogenic genomic variation such as deletions, loss of function, and deleterious missense variants in known disease-causing genes^24^. This leaves the need to be able to reliably detect and prioritize for analysis more complex variation, such as variants impacting gene expression and regulation that may be outside of coding regions (such as in regulatory non-coding and deep intronic regions) as well as complex structural variation such as small copy neutral inversions which may disrupt the association between a gene and its regulatory elements^25^. Although whole-genome sequencing is becoming more widely available diagnostically, and has the potential to detect non-coding, deep intronic, and complex structural variations, many diagnostic laboratories still limit their reporting to variants impacting coding regions of genes and less complex structural variation such as deletions, given robust reporting guidelines are still limited to these types of variants^24,26^.

Cutting-edge ‘omic’ technologies such as long-read and RNA sequencing, which may help better define complex variants and interrogate their impact on gene expression (respectively), are still generally not available outside of the research setting^26,27^.

The number of validated gene-disease associations continues to be made at a frenzied pace^9^, with over 3800 genes included in curated gene lists and so far known to be associated with human disease (the ‘Mendeliome’)^28^. However, we still have far to go to accurately map the potential impacts of deleterious variation in our >20,000 coding genes, let alone the implications of variation in non-coding DNA, for example, long non-coding RNA and antisense RNA^29^.

These limitations in our knowledge can only be overcome with ongoing research and data sharing, including through international collaboration. An undiagnosed diseases program (UDP) is a framework for such research and its integration into clinical care, enabling the discovery of new genotype-phenotype correlations, and more complex genomic variation that may be missed by ‘routine’ diagnostic testing^30^.

All these barriers to a timely and accurate diagnosis are amplified for those living in low- and middle-income countries and/or regional, rural, and remote areas, for those with lower health literacy, and those from culturally and linguistically diverse and indigenous communities^8,31–33^, leading to health inequities that compound those often already present in these communities.

### The importance of global collaboration to shorten diagnostic odysseys

The first formal UDP was established in 2008 at the United States National Institutes of Health (NIH) in Bethesda, Maryland, facilitating integrated clinical and genomic evaluations for people with suspected but undiagnosed rare conditions^34^. Its success in improving diagnostic yield and shortening the time to diagnosis led to the expansion of UDPs across the US within the NIH-funded Undiagnosed Diseases Network (UDN) established in 2014. Soon after, the Undiagnosed Diseases Network International (UDNI) was established following two international conferences in Rome (2014) and Budapest (2015)^35^.

The UDNI now has over 161 members from 55 different countries. Most members are linked to individual UDPs in each continent across the world (except Antarctica) (data provided by UDNI, February 2024). The UDNI is a partnership between clinicians, researchers, and patient organizations. In particular, the Swedish Wilhelm Foundation (WF) has been instrumental in supporting the UDNI and its efforts to shorten the diagnostic odyssey for children, founded by H.C. and M.C. (see Box 1). This partnership ensures that the UDNI’s endeavors are grounded in the needs of individuals and their families with undiagnosed conditions. The WF was established in 2002 by Mikk and Helene Cederroth, and arose from the profound loss of their three dearly beloved children: Wilhelm (1983–1999), Hugo (1991–2002), and Emma (1994–2000), who all had the same undiagnosed disease. The WF’s mission spans a variety of initiatives, including leading a supportive Facebook group for parents navigating undiagnosed diseases in their children. Furthermore, WF arranges family camps tailored to children in Sweden with undiagnosed diseases and their siblings, in addition to hosting biweekly virtual gatherings for parents seeking comfort and guidance. In 2014 the Common Fund, within the Office of the NIH Director, along with the WF sponsored two International Conferences on Rare and Undiagnosed Diseases (Rome, September 29-30, 2014, and Budapest, June 26-27, 2015) which were instrumental in leading to the formation of the UDNI as mentioned above.

The UDNI continues to hold annual International Conferences on Rare and Undiagnosed Diseases, hosted by a different global institute each year. These events include invited talks, presentations, and working meetings. A highlight of the UDNI scientific meetings has been the opportunity for members to share successes and challenges from their individual UDP with a global ‘brains trust’. International and interdisciplinary advice can help each UDP determine which diagnostic and research steps may be appropriate for those remaining undiagnosed.

Another important component of the UDNI is the UDNI Patients Area, which is managed by the WF. Here patients from individual UDP, with appropriate informed consent, can share clinical and diagnostic information, including photographs. This ‘share their story’ area provides an opportunity for diagnostic experts to review and suggest diagnoses^36^.

Box 1 The Wilhelm Foundation’s goal is for all undiagnosed children to be diagnosed Bringing together the leading diagnostic specialists from all corners of the world into a world congress and getting them to cooperate is the Wilhelm Foundation’s hope for solving these mysterious diseases that primarily affect children and adolescents. The Wilhelm Foundation is committed to integrating Diversity, Equity, and Inclusion into everything they do—both in their external work and internally at the Wilhelm Foundation.

To value diversity is to respect and appreciate race; religion; skin color; gender and gender identity; ethnicity; nationality; sexual orientation; physical, mental, and developmental abilities; age; and socioeconomic status.

The Foundation is committed to fair treatment, access, and advancement for all. To help build a more just, inclusive, and equitable future, people from all backgrounds and experiences are embraced. The goal is to build an environment where everyone feels respected and valued, and the beauty in diversity is celebrated.

Visit the Wilhelm Foundation website: https://wilhelmfoundation.org/

*Mikk and Helene Cederroth*

### Establishing the UDNI Diagnostic Working Group

To better pursue the role of the UDNI as diagnostic support for UDPs, the UDNI in 2021 established the Diagnostic Working Group (DWG), to provide a forum and framework for UDP members to seek advice, with the explicit consent of their patients, from other members of the UDNI and their teams.

The DWG is coordinated by its co-chairs (L.B., A.N., and E.E.P.) with bioinformatics support from F.T. The WF is a key partner of the DWG and has supported the establishment of a framework for informed consent for data collection and the establishment of an online secure access Forum for confidential discussions regarding potential diagnoses and data sharing. The above-mentioned public-facing Patients Area (https://www.udninternational.org/en-ricerca_patients#form_ricerca), coordinated by the WF, also allows the UDNI DWG to celebrate when diagnoses have been made.

DWG members meet regularly, synchronously (via videoconference), and asynchronously (via emails and dedicated platforms) to review cases referred by UDNI members for diagnostic consultation. The overall goals of the UDNI DWG (Table 1) are to provide support and advice to local UDPs to (a) accelerate diagnoses for more families; (b) support and share knowledge and skills with developing UDPs, particularly those in lower resource areas; and (c) promote discovery and expand global medical knowledge.

**Table 1:** UDNI DWG goals, strengths, challenges, and opportunities

Goals	Strengths
• Accelerate diagnoses for more families • Share knowledge and skills with developing UDPs, particularly those in lower resource areas • Promote discovery and expand global medical knowledge	• International pool of experts • Educational opportunities • Wide engagement in a range of high-, middle- and low-income countries • Central role of patient organization ensures DWG is grounded in the needs of undiagnosed families • Multiple means for volunteers to interact and participate including in person Hackathons, online forum, and video conferences
Challenges	Opportunities
• Safe and timely sharing of genomic data and samples • Current need for multiple consent processes • Differences in time zones • Volunteer organization, lack of funded administration and technical staff • Lack of UDP and UDNI membership in many countries, especially middle and low income	• Expansion of access to diagnostic technologies and analysis, especially to low- and middle-income countries • A unifying, accessible, and culturally appropriate informed consent process • Broader educational reach, including to countries and regions currently lacking UDP to support the growth of UDPs internationally • Increased engagement with patient groups from a range of countries to improve cultural safety

*DWG* Diagnostic Working Group, *UDNI* Undiagnosed Disease Network International, *UDP* undiagnosed diseases program

Underlying the activities of the UDNI DWG is support for equitable access to a timely and accurate diagnosis for families globally. One strategy has been to connect established and well-resourced UDPs with newly established UDPs. For example, the Penelope Program at the University of Utah, US, and the UDP at the Karolinska Institute in Stockholm, Sweden, have connected with newly established UDPs including the GeneAdd program at the Sydney Children’s Hospitals Network, Sydney, Australia, and UDPs in low- and middle-income countries including The Aga Khan University, Karachi, Pakistan; Ga-East Municipal Hospital, Accra, Ghana; University of Kinshasa, Democratic Republic of Congo; and Faculty of Medicine and Dentistry at the University of Bamako, Mali.

**Fig. 1:**
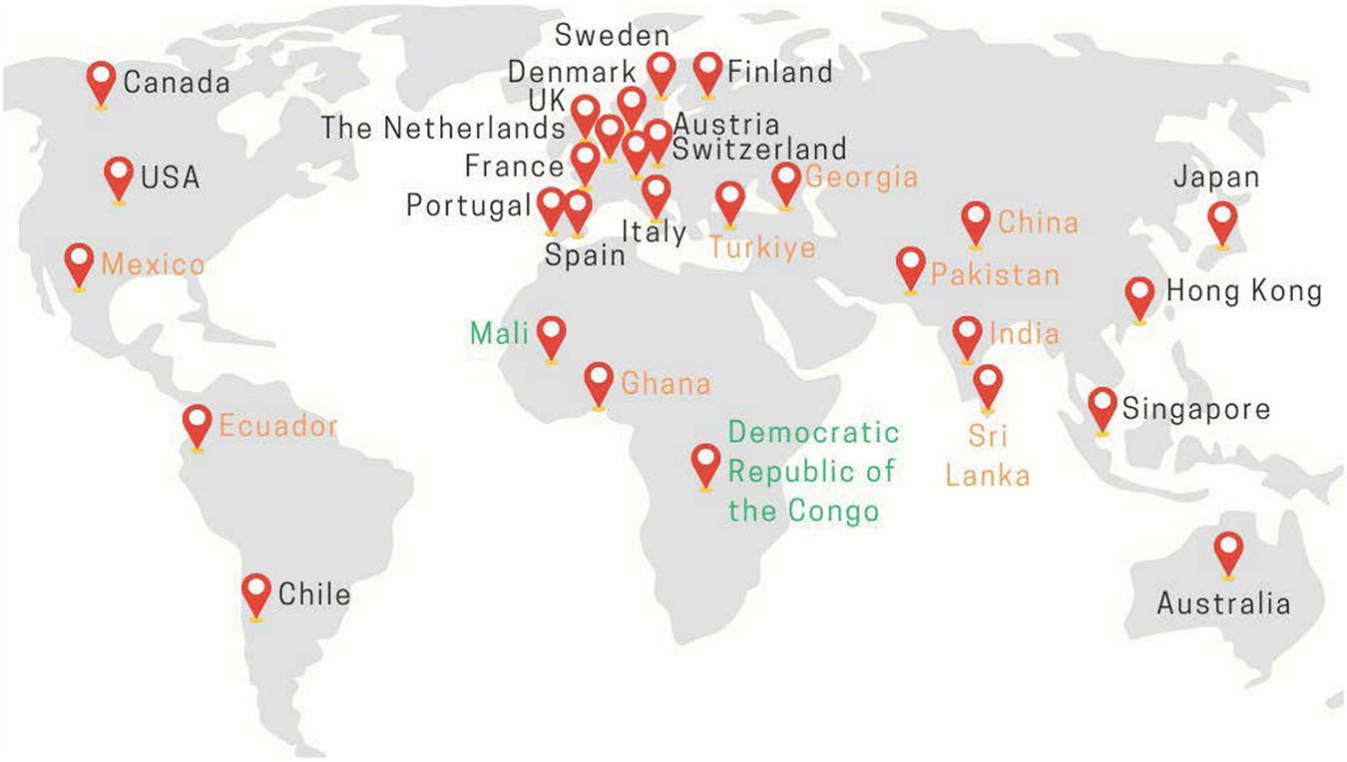
Locations of UDNI DWG members. UK, United Kingdom; USA, United States of America; Black font indicates high-income countries, orange font indicates middle-income countries and green font indicates low-income countries (based on incomes reported by the World Bank as of February 2024). Figure created using world map pin timeline template ©Rizelle Anne Galvez via Canva.com.

UDNI members continue to generously donate their time and resources to support the global goals of the DWG. The number of volunteer members continues to grow and includes professionals (clinicians and scientists) from a range of clinical specialties, diagnostic laboratories, and research groups. When last surveyed in December 2023, the group included 86 members from 29 countries across Europe (42 members), North America (18 members), Asia (14 members), the Pacific region (5 members), Africa (5 members), and South America (2 members) (Fig. 1).

Members are interdisciplinary including health professionals with an interest in finding diagnoses for those with rare diseases (60%), molecular genomics experts (19%), bioinformaticians (9%), functional genomics experts (2%), other types of rare disease researchers (2%) and M.C. and H.C., the patient advocacy leads from the WF. Of the health professionals, the majority (54%) are clinical geneticists, but expertise is multidisciplinary and includes neurologists (9%), pediatricians (5%), neonatologists (9%), dermatologists (3%), endocrinologists (3%), radiologists and neuroradiologists (4%) and a surgeon, nephrologist, psychologist and genetic counselor. New members are welcome with contact details for the co-chairs on the UDNI website (https://www.udninternational.org/).

The DWG typically meets monthly via videoconference to discuss 2-3 undiagnosed families. The structure of the DWG has been iteratively improved to address the challenges of data sharing in a safe and time-sensitive manner, and to enhance the likelihood of making a diagnosis without an undue time burden on the members (all unpaid volunteers).

### The patient and clinician journey through the UDNI DWG

**Fig. 2:**
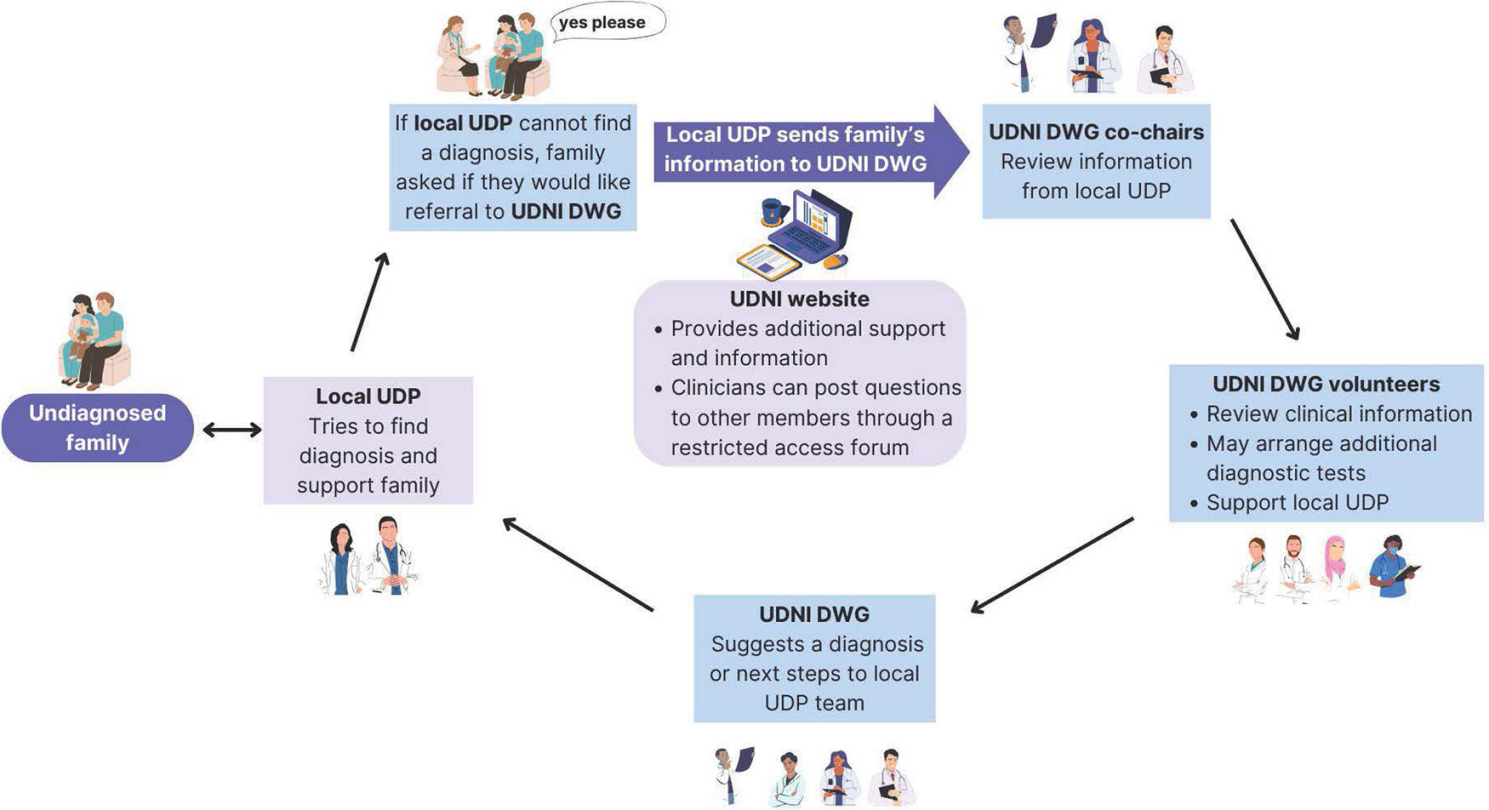
Patient journey through the UDNI DWG. *DWG* Diagnostic Working Group, *UDNI* Undiagnosed Diseases Network International, *UDP* undiagnosed diseases program. Figure created via Canva.com.

The current patient and clinician journey through the UDNI DWG program is shown in Fig. 2. Families who could not be diagnosed through their individual programs are proposed to the DWG co-chairs by clinicians from UDNI-member UDPs. The local team then submits detailed clinical and genomic information from an undiagnosed family to the UDNI forum, a private secure online platform managed by the WF. In addition to capturing detailed data about individuals and their families, the forum facilitates asynchronous participation by DWG volunteers who cannot join the monthly sessions due to time zone clashes or work commitments.

Local teams present a phenotypic and diagnostic summary at a UDNI DWG meeting. Volunteers review the available information and then post potential differential diagnoses or, during a meeting, suggest possible diagnoses and next research steps. Support from DWG members has included a reanalysis of existing genomic data and new multi-omics sequencing. This process facilitates the sharing of both knowledge and resources among the members. In addition to the forum, a public-facing summary is uploaded into the Patients Area of the UDNI.

The DWG does not directly interact with the families. This is the primary responsibility of the referring (local) team, which keeps in close contact with the family and keeps them up to date with progress and any diagnoses. Once achieved, diagnoses are celebrated by the placement of a ‘Diagnosed’ banner across the individual in the Patients Area, as well as a short summary and relevant patient information about the diagnosis and available support groups. As a result, the UDNI DWG facilitates education and equity of access to the most advanced diagnostic technologies for all members.

### Iterative improvement in the UDNI DWG and future directions

**Fig. 3:**
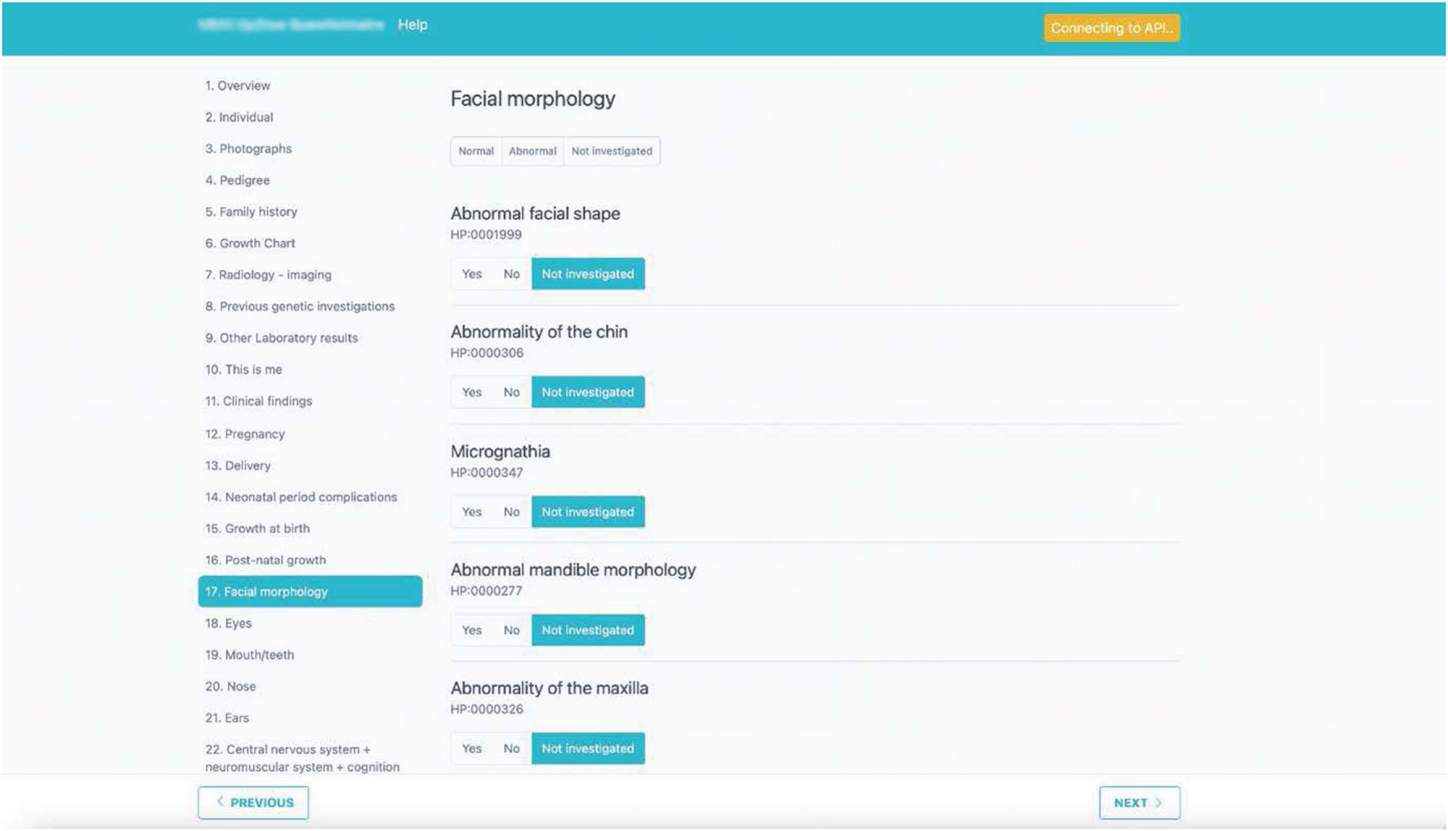
The tip2toe form. Reproduced with permission from Ann Nordgren.

A useful initiative has been the creation of a detailed phenotypic form ‘tip2toe’ (tipp being the Swedish word for top) (Fig. 3) which was designed by the Karolinska Undiagnosed Disease Program team, led by A.N., and user-tested by the co-chairs. The form facilitates uniform data collection with sufficient detail for effective diagnostic review in a time-efficient, simple, and easily navigable format.

In the ‘tip2toe’ form, phenotypic information is collected in human phenotype ontology (HPO) terms, which enables standardized data collection that can be integrated into genomic diagnostic bioinformatic platforms^37^. However, HPO terms do not yet capture the evolution of symptoms and clinical features over time, critical for a comprehensive rare disease diagnostic evaluation. Therefore additional ‘tip2toe’ sections capture these critical narrative accounts, clinical photographs, and key results, including imaging and findings from genomic investigations. The tip2toe form is now published and available to all UDNI members, and the program code available as open source for use or modification (https://tip2toe.org/).

The UDNI DWG collaborates closely with other UDNI working groups, including data sharing and technology, genetic counseling, and low- and middle-income countries groups^38^. Timely sharing of genomic data and samples remains a challenge. The UDNI website includes a consent form for the sharing of personal data (including health and health data, photographs, and genetic data for the purpose of finding a diagnosis for a rare disease), which is the co-responsibility of the WF and the Istituto Superiore Di Sanita, Italy, and needs to be signed by the patient or their legal guardian in order to be enrolled in the UDNI DWG. However, although this form grants permission for sharing of phenotypic and diagnostic information on the UDNI website, the legal-regulatory requirements in different countries mean that the sharing of genomic data and/or samples between a submitting UDP and a member of the UDNI DWG typically requires additional consent forms and processes at both sites. There are many logistical challenges to sharing data and samples between countries if not continents, with precise shipping requirements for samples and alignment of bioinformatic frameworks. These processes can be long and complex, delaying access for families to the resources of the DWG. A more streamlined process that balances the individual autonomy of families with the ethical and governance requirements of individual UDP members would be extremely beneficial^18^.

In addition to the ‘tip2toe’ module, another initiative to provide accessible and family-centered information about diagnosed conditions includes a collaboration with the UDNI genetic counseling group to pilot family-friendly templates for results and clinical summaries, co-designed with patient and clinician input. It will be critical to ensure these are easy to understand for those with lower health literacy and can be translated into the relevant language by the local UDP.

In 2023, the UDNI DWG supported the first Undiagnosed Hackathon. The Hackathon was arranged by the WF, Karolinska Institutet, Karolinska University Hospital, and the software company Phenotips. Around 100 clinicians and researchers from 28 countries worked together over 48 h at the Karolinska Institute, Stockholm, to try to ‘solve’ 13 undiagnosed individuals from 10 families (three families having two similarly affected siblings) from four continents who, prior to the event, had been carefully phenotyped and sequenced with comprehensive whole-genome analysis utilizing both long-read and short-read DNA and RNA sequencing. The UDNI DWG facilitated preparatory case-based discussion ahead of the Hackathon and continued with post-Hackathon diagnostic discussions. To date, a molecular diagnosis has been confirmed for four previously unsolved patients from four countries, and further investigations are ongoing with several individuals having a promising variant in genes not previously associated with a monogenic condition. It is hoped that the combination of in-person intensive Hackathons, online synchronous UDNI DWG meetings, and the online forum will combine to drive innovation and education to meet the aims of the DWG most efficiently.

It is acknowledged that the UDNI DWG, and the UDNI more broadly, sit within the context of a welcome growing number of national genomic testing programs that incorporate research, many applying innovative approaches to reach diagnoses for those most in need as quickly and equitably as possible, such as the national neonatal intensive care genomics Baby Bambi project in Israel and the acute care genomics project in Australia^39,40^ and regional and international networks and collaborations (such as the European SOLVE-RD and the International Rare Diseases Research Consortium [IRDiRC])^30,41^. Such networks are advancing much-needed collaborations and innovative technologies to shorten the diagnostic odyssey and improve diagnostic yields, but we still have far to go to bring equitable access to diagnoses globally. Dedicated sponsorship or grant funding to support new sequencing and reanalysis would likely benefit more families and more UDPs, especially in low-resource regions. Other such opportunities along with a summary of the UDNI DWG goals, strengths, and challenges are outlined in Table 1. For now, the generosity of current volunteers and the WF provides a shining example of the power of goodwill and global collaboration to improve equity of access to diagnoses for all families living with undiagnosed rare diseases, regardless of their economic, social, or geographic challenges, and in line with the goal of universal health coverage emphasized in the UN resolution^1,41^.

## Data Availability

Data sharing is not applicable to this article as no datasets were generated or analyzed during the current study.
